# Effort During Ethanol Breath Testing Impacts Correlation with Serum Ethanol Concentration

**DOI:** 10.5811/westjem.24998

**Published:** 2025-02-06

**Authors:** Samuel J. Stellpflug, William H. Menton, Bjorn C. Westgard, Ryan D. Johnsen, Alexander M. Coomes, Robert C. LeFevere, Michael D. Zwank

**Affiliations:** * Regions Hospital, Department of Emergency Medicine, Saint Paul, Minnesota; † VA Healthcare System, Minneapolis, Minnesota

## Abstract

**Introduction:**

The gold standard for quantifying ethanol intoxication in patients is serum testing. However, breath testing is faster, less expensive, and less invasive. It is unknown whether perceived effort during a breath ethanol test impacts the accuracy of the test and the correlation with serum concentration. In this study we analyzed whether perceived “poor” effort during breath ethanol testing would result in worse correlation than perceived “normal” breath-testing effort with respect to serum ethanol concentration.

**Methods:**

Subjects were identified retrospectively over a 49-month period if they had both a breath ethanol test and a serum ethanol test obtained during the same ED visit within 60 minutes of each other, if they had their effort during the breath test recorded as “normal” or “poor” by the person administering the test, and had non-zero breath and serum ethanol concentrations. We completed descriptive and correlation analyses.

**Results:**

A total of 480 patients were enrolled, 245 with normal and 235 with poor effort. The patients with normal breath-test effort had mean breath and serum concentrations of 0.19 grams per deciliter (g/dL) and 0.23 g/dL, respectively. The patients with poor breath-test effort had mean breath and serum concentrations of 0.19 and 0.29 g/dL, respectively. The correlation coefficient between breath and serum ethanol values was 0.92 (95% confidence interval (CI) 0.84–0.96) for good effort and 0.63 (95% CI 0.53–0.74) for poor effort.

**Conclusion:**

The assessment of breath exhalation effort is meaningful in determining how well a patient’s breath ethanol level correlates with the serum ethanol concentration. Poor breath effort, when compared to normal breath effort, was associated with higher ethanol levels as well as a larger difference and a greater variability between breath and serum values. If an accurate ethanol level is important for clinical decision-making, a physician should not rely on a poor-effort breathalyzer value.

## INTRODUCTION

Breath testing for ethanol has been discussed in medical literature for nearly 150 years.^1^ Ethanol testing is often used in emergency departments (ED) and has historically included blood testing, breath testing, or both. Breath testing has been used as a surrogate for the gold standard serum testing and has distinct advantages over blood: it is faster, less invasive, and less expensive.^2^ Despite longstanding study of the topic and wide acceptance of its use, it has not been well established whether a poor expiratory effort, as judged by the tester, affects the accuracy of the test.

When administering a breath ethanol test, the operators of the device will often comment on the expiratory effort of the patient. The inference is that an effort deemed “poor” by the tester will not be as accurate as a “normal”-appearing expiratory effort. The impact of apparent exhalation effort on the correlation between breath and serum levels has not been clearly established. Clarifying this could impact patient care and could provide utility in forensic evaluation. Our primary objective in this study was to determine whether a patient’s expiratory effort, as perceived by the tester, affected the breath ethanol test results when compared to serum. Secondary objectives included determining overall correlations between breath and blood testing within a single hospital encounter.

## METHODS

This study was approved by the Health Partners institutional review board. A retrospective electronic health record (EHR) inquiry was performed to include all patients over a 49-month period who had breath ethanol testing with documented perceived exhalation effort (“normal” or “poor”) and serum ethanol testing completed during a single ED visit at a large, tertiary-care hospital. At this hospital, the individual performing the breath test, typically an emergency medicine technician or registered nurse, chooses one of these two effort categories as an electronic checkbox when entering the ethanol value into the EHR. The assessment of effort is done using their own clinical judgment. All breath ethanol tests were performed using the Alco-Sensor FST (AlcoPro Inc, Knoxville, TN). All serum ethanol tests were done using the ARCHITECT c8000 (Abbott Laboratories, Abbott Park, IL).

Data collected for this study included the following: time of breath ethanol test; the patient’s perceived breath testing effort; the result of the breath ethanol test; the time of the blood draw for serum ethanol testing; and the result of the serum ethanol test. Subjects were included if they had both a blood and breath ethanol test done within a 60-minute time interval. Subjects were excluded if either the breath or serum concentration was 0 grams per deciliter (g/dL). This was done because some of the blood draw and breath tests had enough time between them in the same subject such that a 0 g/dL value may have inaccurately impacted the correlation calculations, as the patient may have naturally reached a level of 0 g/dL well before the second test occurred.

### Statistics

The associations between breath and serum ethanol levels, controlling for breath effort, appeared highly linear on initial graphical visualization of the data. Therefore, the relationships between these variables were explored further using a combination of zero-order Pearson correlations and linear regression. We examined the properties of breath ethanol concentrations, serum ethanol concentrations, and the associations between them.

## RESULTS

A total of 480 subjects were included in the study. Of these subjects, 245 showed normal effort and 235 were documented as poor effort. Additionally, 237 patients had a time interval of less than 15 minutes between breath and serum values, 112 had a time interval of 16–30 minutes, and 131 had a time interval of 31–60 minutes. There were 184 patients who had blood drawn before the breathalyzer and 288 who had blood drawn after the breathalyzer; eight patients were tested concurrently. Among all patients, the mean breath ethanol was 0.19 g/dL, while the mean serum ethanol was 0.26 g/dL. The patients with normal breath test effort had mean breath and serum concentrations of 0.19 g/dL and 0.23 g/dL, respectively. The patients with poor breath test effort had mean breath and serum concentrations of 0.19 and 0.29 g/dL, respectively. The correlation coefficient between breath and serum was 0.92 (95% confidence interval [CI] 0.84-0.96) with normal effort and 0.63 (95% CI 0.53-0.74) with poor effort. Descriptive results and correlation analysis between the tests are presented in the Table. A plot displaying individual breath and serum values, as well as lines of best fit by effort group, is presented in the Figure.

**Figure.**
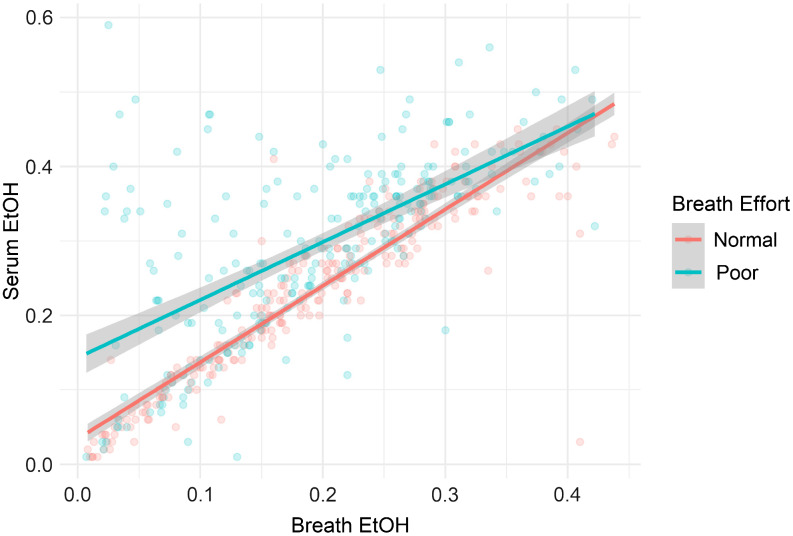
Plot of breath and serum ethanol values and lines of best fit for “normal”- and “poor” effort groups.

## DISCUSSION

Our primary objective in this study was to determine whether the perceived level of expiratory effort during a breath ethanol test impacts the accuracy of the breath test when compared to a serum ethanol test. We documented the subject’s effort as perceived by the tester because it reflects a common assessment in the clinical setting. Clinicians are often given the result of the breath test along with the assessor’s subjective description of the breath effort.

The results of this study indicate that the assessment of breath exhalation effort is meaningful in determining how well a patient’s breath ethanol level correlates with their serum ethanol concentration. While breath ethanol values were generally lower than serum ethanol values (regardless of effort), this difference was both greater and more variable among patients with poor effort. This is shown by a greater difference in values for those patients with poor effort (Table), by a higher standard deviation in difference values, and by a lower correlation coefficient in this group (Table and Figure). This is consistent with prior findings in a study by Gibb et al who examined whether “cooperativeness” with the breathalyzer was associated with differences in breath vs serum values.^2^ Cooperation was defined as whether a patient “understood and followed through with the instructions to perform a smooth, forced expiration into the analyzer.” While this was an informative study, in practice, documentation is related to effort and not to cooperation. Thus, our study is a more practical assessment of real-world experience.

**Table.** Patient ethanol levels (breath and serum; grams per deciliter) and correlation coefficients.

	Both effort groups	Normal effort	Poor effort
Number of patients	480	245	235
Mean breath EtOH	0.19	0.19	0.19
Mean serum EtOH	0.26	0.23	0.29
Mean EtOH difference within individual patients [95% CI]	−0.07 [0.09, −0.23]	−0.04 [0.04, −0.12]	−0.10 [0.10, −0.30]
Correlation coefficient between breath and serum [95% CI]	0.75 [0.7, 0.82]	0.92 [0.84, 0.96]	0.63 [0.53, 0.74]

*EtOH*, ethanol; *CI*, confidence interval.

The “poor effort” group also demonstrated substantially higher serum alcohol concentrations than the “normal effort” group (0.29 g/dL vs 0.23 g/dL). This is perhaps unsurprising and suggests possibly reduced ability to coordinate a good expiratory effort or less motivation to participate in testing. We did not extend the analysis past a 60-minute interval between breath and serum tests because any conclusions beyond this time frame were not felt to be clinically applicable. Analysis of subjects with a narrow time difference between breath and serum testing is important to minimize any possible impact of ongoing ethanol metabolism between execution of the different testing modalities.

## LIMITATIONS

Assessment of patient expiratory effort in breath ethanol testing is a subjective measure. However, it is the same subjective measure assessed during real patient care. More formal measurement of expiratory capacity could add perspective and potentially accuracy as well. In addition, given the retrospective observational nature of our data, breath testing and blood samples for serum testing were often not performed simultaneously. We did use a narrow time frame for analysis, thus negating much metabolism. A prospective study obtaining blood samples for serum ethanol testing at the time of breath ethanol testing would be necessary to eliminate this potential confounder. Finally, while the breathalyzer used at our hospital is a commonly used device, other devices may be used elsewhere, and their measurement properties may vary.

## CONCLUSION

Breath ethanol concentrations were generally lower than serum ethanol concentrations. Poor exhalation effort on breath ethanol testing correlated with a larger difference between breath and serum ethanol concentrations and with greater variability in the difference between the two. This can be relevant in clinical and forensic settings.
